# Glutamate, Immune Cells and Alzheimer’s Disease: An Attempt to Link the Triad

**DOI:** 10.1007/s10571-026-01784-w

**Published:** 2026-07-28

**Authors:** Nikolaos A. Chinas, Harry Alexopoulos

**Affiliations:** 1 Department of Cell Biology and Biophysics, Faculty of Biology, National and Kapodistrian University of Athens, Athens, Greece; 2 Department of Genetics, Microbiology and Statistics, Institut de Biomedicina de la Universitat de Barcelona (IBUB), Universitat de Barcelona, Barcelona, Spain

**Keywords:** Neurochemistry, Glutamate, Synapses, Human immune cells, Neurodegeneration, Neuro-immuno modulation

## Abstract

Glutamate (Glu), although the main excitatory neurotransmitter (NT) of the central nervous system (CNS), evidence recognizes its role in influencing immune cell function and activation, including cytokine production, cell proliferation, migration, and survival. Importantly, dysregulation of Glu homeostasis has been implicated in the pathogenesis of Alzheimer’s disease (AD), a neurodegenerative disorder characterized by progressive cognitive decline, neuronal loss and neuroinflammation. This review synthesizes current evidence on the biochemistry of the glutamatergic synapse, as well as the expression and signaling of Glu receptors (GluRs) in human immune cells. Furthermore, it examines two major hypotheses of AD pathogenesis —the glutamatergic and the neuroimmune hypothesis— and proposes a unified glutamatergic–neuroimmune framework. In this model, disrupted glutamatergic signaling at the neuron–glia–immune interface may represent a central contributor to AD pathology. Understanding the glutamatergic–neuroimmune axis may provide novel insights into therapeutic strategies targeting neuroimmune modulation in AD.

## Introduction

In the past, the nervous system was thought to be an immunoprivileged site, isolated from immunity and that was attributed mainly to the blood-brain barrier (BBB) (Nutma et al. 2019; Louveau et al. 2015). However, the previous century proved that nervous and immune systems regulate each other’s responses through a wide array of molecules, such as cytokines and neurotransmitters (NTs), after binding to their respective receptors (Mapunda et al. 2022; Nutma et al. 2019). Immune cells can infiltrate brain parenchyma and secrete different pro-inflammatory and anti-inflammatory cytokines which influence neuronal activity (Mapunda et al. 2022). For example, the anti-inflammatory cytokine interleukin-4 (IL-4) activates interleukin-4 receptor α chain (IL-4Rα) expressed on sensory neurons and mediates chronic itch (Mahmoud et al. 2023). Similarly, interferon-γ (IFN-γ) secreted by infiltrating T cells in the subventricular zone (SVZ), has been shown to inhibit the proliferation of neural stem cells (NSCs) (Cutler and Kokovay, 2020), highlighting the direct regulatory impact of immune-derived molecules on neuronal populations.

The view has shifted more significantly, as data from neurodegenerative and autoimmune disease research have revealed widespread immune activity across virtually all compartments of the central nervous system (CNS) (Mapunda et al. 2022; Nutma et al. 2019). In multiple sclerosis (MS), for instance, B cells produce autoantibodies (auto-Abs) against myelin components both within the cerebrospinal fluid (CSF) and intrathecally, contributing to demyelination and neuronal damage (Chinas et al., 2025). Growing evidence also implicates immune dysregulation, in the pathogenesis of neurodegenerative diseases such as Alzheimer’s disease (AD) and Parkinson’s disease (PD). These conditions are characterized by the progressive loss of neuronal populations mainly in the hippocampus in AD and in the substantia nigra in PD affecting memory and movement, respectively (Netzahualcoyotzi et al. 2024). Additionally, it has been recently characterized the glymphatic system, where immune cells surveil meninges for potential threats, such as pathogens, and sequester them into the cervical lymph nodes (Jessen et al. 2015).

All these examples illustrate the diverse and significant ways immune cells influence neuronal integrity and function. However, the reverse relationship —how neurons and glial cells modulate immune responses— is not well established. A central mechanism in this bidirectional communication involves NTs [e.g., dopamine (DA), serotonin (5-HT)] (Hodo et al. 2020) and gliotransmitters (GTs) (e.g., D-serine (D-Ser), adenosine triphosphate (ATP)] (Sahlender et al. 2014), the chemical messengers released by neurons and glial cells, respectively, to communicate through autocrine and synaptic manners (Hodo et al. 2020; Sahlender et al. 2014).

Remarkably, studies have shown that immune cells not only express receptors for various NTs but can also synthesize and release them themselves (Franco et al. 2007) [9]. For example, leukocytes express active L-Dopa decarboxylase (DDC), the enzyme that catalyzes the conversion of L-3,4-dihydroxyphenylalanine (L-dopa) to DA and 5-hydroxytryptophan (5-HTP) to 5-HT (Kokkinou et al. 2009). This aspect challenges the view of the nervous and immune systems as separate entities and instead supports the concept of their co-evolution, particularly in the light of their shared similarities as they are analogous systems for sensing and responding to the environment. Firstly, they communicate with soluble molecules (cytokines or NTs) that enable fast, bidirectional and long-distance communication. In addition, both systems form specialized cell-cell structures (chemical: see below and *immunological synapse*: an antigen-presenting cell with a T-cell) that transfer information. Moreover, both systems are characterized by plasticity and memory, meaning they can adapt based on prior experience and respond more efficiently upon re-stimulation as it can been seen with long-term potentiation (LTP) and immunity. They also share mechanisms of cell migration and guidance, whereby cells response to chemical cues (e.g., chemokines and semaphorins) to achieve precise spatial organization and coordination of responses. At the systems level, both systems consist of highly diverse cell populations arranged in complex networks, capable of processing information and generating coordinated outputs. Finally, they share common receptors (e.g., plexins), signaling pathways [e.g., nuclear factor κ B (NFκΒ)] and gene regulation processes (e.g., allelic exclusion) during their development, raising the possibility that the two systems may have evolved from a common ancestral cell type (Kioussis et al., 2009).

Over the years, there have been reports of the dual role of transmitters, such as acetylcholine (Ach), DA or γ-aminobutyric acid (GABA), in the regulation of immune responses in both normal (e.g., neurodevelopment, synaptic plasticity) and disease-context conditions (e.g., schizophrenia, epilepsy, AD, PD, MS) (Ng et al. 2026; Li et al. 2025; Franco et al. 2007). Among them, glutamate (Glu), the most abundant excitatory transmitter in the CNS, is emerging as a key modulator of immune cell behavior depending on context and receptor subtype (Ng et al. 2026; Franco et al. 2007).

In this review, we provide a summary of chemical transmission and emphasize on the biochemistry of the glutamatergic synapse. Also, we briefly describe the immune system and integrate up-to-date evidence of the expression and signaling of Glu receptors (GluRs) in human immune cells, Finally, we discuss two major frameworks of AD pathogenesis —the glutamatergic and the neuroimmune hypothesis—and propose a unified glutamatergic–neuroimmune model, in which the two hypotheses converge into a single, integrated perspective.

## Chemical Transmission

Otto Loewi, first demonstrated in 1921 that nerve impulses can be transmitted chemically (Valenstein 2002). Chemical transmission is the most significant way of neuronal communication with highly regulated yet flexible events that provide the basis for neuroplasticity (Ahmed et al. 2026).

Synapses are highly specialized contact points between neurons that facilitate both the transfer and processing of information, organizing neurons into extensive, overlapping, and intricately connected networks. Their development, functional features, and adaptability is driven by an underlying molecular system governed by a diverse array of signaling molecules acting across the synapse (Südhof, 2017).

NTs can be excitatory (e.g., Glu), inhibitory (e.g., GABA) and modulatory (e.g., DA) depending on their effect on the postsynaptic neuron. Excitatory ones, cause depolarization [calcium (Ca^+ 2^) or/and sodium (Na^+^) ions influx], whereas inhibitory NTs induce hyperpolarization [chlorine ions (Cl^−^) influx or/and potassium ions (K^+^) efflux) (Ahmed et al. 2026). Also, they can be further classified into amino acids (e.g., Glu), monoamines (e.g., DA), peptides (e.g., somatostatin), purines (e.g., ATP), gases (e.g., NO) and others (e.g., Ach) based on their chemical structure. (Chemchem et al. 2022). All the above categories can be broadly classified as canonical NTs. However, steroids and D-aspartic acid (D-Asp) gain ground as noncanonical NTs due to their participation in both homeostatic and pathological (e.g., AD) conditions (Teleanu et al. 2022).

Neurotransmission begins with the synthesis of NTs by specific enzymes either in the cell body (neuropeptides) or in the presynaptic nerve terminal (classical). These molecules are stored in acidic synaptic vesicles, which protect them from degradation and allow their rapid release upon depolarization of the presynaptic membrane. Depolarization activates voltage-gated calcium channels (VGCCs) enabling the rapid influx of Ca^+ 2^ and subsequent increase of intracellular Ca²⁺ concentration. This triggers the docking and exocytosis of synaptic vesicles at specific sites called active zones, releasing the NT into the synaptic cleft, the narrow space between the presynaptic and postsynaptic neuron (Kovács 2004; Sudhof, 2004). Following exocytosis, synaptic vesicle membrane is recycled via several possible mechanisms of clathrin-mediated endocytosis or clathrin independent fast reuse, the most common named “kiss-and-run” (Sudhof, 2004). Other mechanisms include ultrafast endocytosis (Imoto and Watanabe 2025) and activity-dependent bulk endocytosis (Kokotos et al. 2018).

Following exocytosis, synaptic vesicle membrane is recycled maybe via clathrin-mediated endocytosis or fast reuse named “kiss-and-run” (Sudhof, 2004).

The released NTs interact with receptors in the postsynaptic membrane that fall into two main categories: ionotropic receptors, which directly couple with ion channels, and metabotropic receptors, such as G-protein-coupled receptors (GPCRs), which act through second messengers (Sudhof, 2004). G proteins are heterotrimeric (α, β, γ) and classified into four families according to their α subunit: G_i/o_, Gs, G_12/13_ and G_q/11_ (Zhang et al. 2024a, 2024b).

Termination of neurotransmission is achieved by different ways depending on the NT. DA, noradrenaline (NA), Glu and GABA can undergo reuptake via Na⁺-dependent transporters located either on presynaptic terminals or astrocytes (Kovács 2004; Sudhof, 2004; Zahniser and Doolen 2001). However, astrocytes do not handle all NTs uniformly. For instance, noradrenergic signaling in astrocytes occurs mainly through α_1_-noradrenergic receptors (α1-NARs) activation, inducing Ca^+ 2^ transients that modulate astrocytic activity. In turn, this activity will either “turn-off” or boost the effects of NA on neurons (Wahis et al., 2021).

Moreover, there are NTs such as ACh and neuropeptides that degrade in the synaptic cleft enzymatically [e.g., acetylcholinesterase (AChE)] (Kovács 2004; Zahniser and Doolen 2001). Notably, NT receptors are also present in the presynaptic nerve terminal membrane enabling feedback modulation of NT release as described below for Glu. The presynaptic receptors can inhibit future exocytosis, thus adjusting synaptic strength dynamically (Kovács 2004; Miller 1998).

Lastly, in contrast to small synaptic vesicles, neuropeptides are stored in larger dense-core vesicles and transported into the nerve terminal by axonal transport. The exocytosis of peptidergic granules takes place in sites distinct from active zones and requires multiple or high-frequency stimulation (Russo, 2017; Kovács 2004). Therefore, neuropeptides have long-lasting effects on postsynaptic neurons (Russo, 2017).

## Glutamatergic Synapse

The view of synapse as a site of communication only between a presynaptic terminal and a postsynaptic spine, has shifted towards “the tripartite synapse”. The tripartite synapse incorporates astrocytic processes, leading to a dynamic crosstalk between neurons and astrocytes. The latter not only support metabolically the former or clear the excessive NTs, (Perea et al. 2009) but by releasing GTs —after Ca^+ 2^ intracellular increase— can potentiate (e.g., Glu, D-Ser) or inhibit (e.g., ATP) synaptic transmission (Harada et al. 2016). More recently, this framework has been extended to the “quad-partite synapse” incorporating microglia as an additional key component (Schafer et al. 2013). In this context, microglia play an active role in synaptic refinement and selection, further highlighting their importance in shaping neural circuits (Faust et al. 2025).

**Fig. 1:**
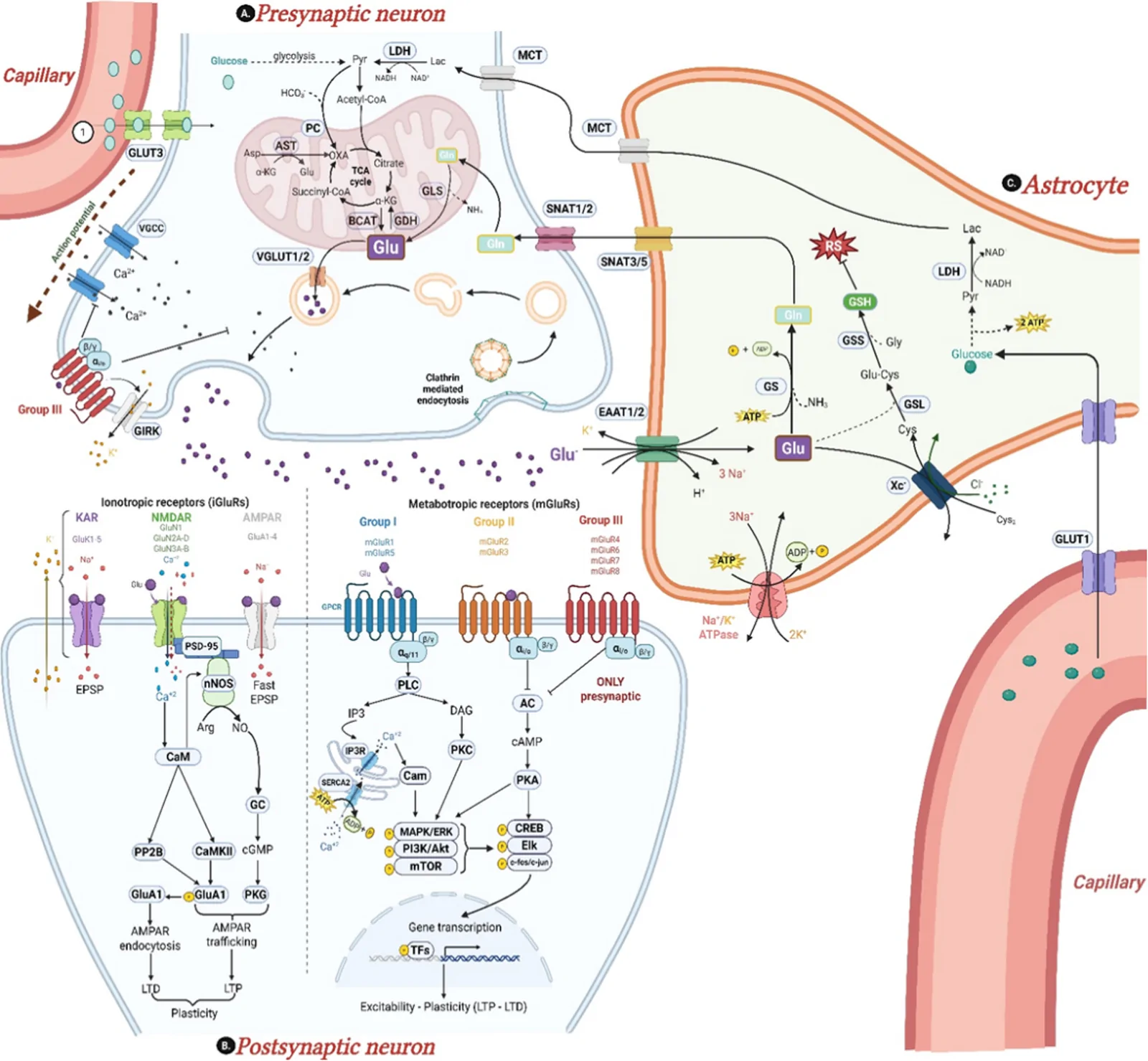
The biochemistry of the glutamatergic synapse. **A** Presynaptic neuron: Within the presynaptic neuron, mitochondrial metabolism serves as a central hub that provides the substrates required for Glu synthesis through the TCA cycle. Newly synthesized Glu is packaged into synaptic vesicles and released into the synaptic cleft in response to Ca²⁺ influx during neuronal firing. **B** Postsynaptic terminal: The released Glu binds to iGluRs or mGluRs on the postsynaptic membrane, initiating intracellular signaling cascades. These cascades typically involve kinase-driven phosphorylation events that ultimately lead to LTP or LTD, depending on the receptor subtype activated. **C** Astrocyte: The astrocyte provides essential metabolic support to the synapse. It regulates extracellular Glu levels through the Glu/Gln cycle, contributes to antioxidant defense via GSH synthesis, and supplies metabolic fuel through Lac production. *Glu* glutamate, *TCA* tricarboxylic acid, *Gln* glutamine, *iGluRs* ionotropic glutamate receptors, *mGluRs* metabotropic glutamate receptors, *LTP* long-term potentiation, *LTD* long-term depression, *GSH* glutathione, *Lac* lactate

A detailed illustration of the glutamatergic synapse is provided in Fig. 1.

### Glutamate Synthesis, Storage, Release

Glu is the dominant NT in the CNS with a great number of synapses being glutamatergic (Gasiorowska et al. 2021; McKenna 2007). Glucose enters the presynaptic neuron via glucose transporter 3 (GLUT3) and is metabolized via glycolysis into pyruvate (Pyr). Pyr is then converted into acetyl coenzyme A (acetyl-CoA), which fuels the tricarboxylic acid (TCA) or Krebs cycle in mitochondria (Peng et al. 2021; Andersen et al. 2021). Within this cycle, the intermediate α-ketoglutarate (α-KG) provides the carbon backbone for Glu. Through the action of branched-chain amino acid aminotransferase (BCAT), α-KG accepts an amino group (NH_2_) to form Glu (transamination). Amino donors include branched-chain amino acids (BCAAs), such as leucine (Leu), isoleucine (Ile), and valine (Val). The accumulation of Glu in the brain is also due to the highly active aspartate aminotransferase (AST) which uses aspartate (Asp) as an important NH_2_ reservoir for Glu synthesis. AST aminates α-KG forming Glu and another TCA cycle intermediate, oxaloacetate (OAA) which leads to citrate formation and thus TCA cycle ongoing. Although glutamate dehydrogenase (GDH) can remove NH_2_ from Glu, regenerating α-KG and releasing ammonia (NH_3_), the α-KG loss is higher and can result in TCA cycle stall and energy-ATP production will stop. Therefore, pyruvate carboxylase (PC) catalyzes the carboxylation of Pyr to OAA (anaplerosis), fueling TCA cycle (Andersen et al. 2021).

Once synthesized, Glu is transported into synaptic vesicles by vesicular glutamate transporters (VGLUTs). These transporters operate as proton (H^+^)/Glu antiporters, driven by a H^+^ gradient established by vacuolar-type (V-type) H⁺-ATPase: H^+^ accumulate in the lumen of the vesicle, which becomes positive relative to the cytosol. The acidic vesicular lumen and the different membrane potential from that of cytosol provide the energy needed for Glu accumulation (Andersen et al. 2021). Different populations of glutamatergic neurons express either VGLUT1 or VGLUT2 (Fremeau et al. 2004).

Glu release is facilitated by the Ca²⁺ influx that VGCCs mediate while the vesicle membrane is recycled for future Glu loading (Kovács 2004; Sudhof, 2004).

### Glutamate Receptors (GluRs) and Signaling

Released Glu diffuses across the synaptic cleft and activates receptors in the postsynaptic membrane. Glu has both ionotropic (iGluRs) and metabotropic (mGluRs) receptors, which are spread across the postsynaptic density (PSD). PSD protein 95 (PSD95), a major scaffolding protein of the PSD, couples both iGluRs and mGluRs to intracellular signaling systems, sometimes highly interlinked, which regulate LTP and long-term depression (LTD). LTP and LTD are manifestations of synaptic plasticity and require protein synthesis, driven by transcription of specific genes regulated by transcription factors (TFs) (Reiner et al., 2018).

#### Ionotropic Glutamate Receptors (iGluRs)

iGluRs are organized into three main classes, originally named after selective agonists and they include N-methyl-D-aspartate receptor (NMDAR), α-amino-3-hydroxy-5-methyl-4-isoxazolepropionic acid receptor (AMPAR), and kainate receptor (KAR). Most of these receptors in the brain are heteromeric complexes and conduct influxes of Na^+^ and Ca^+ 2^ and efflux of K^+^, but ion selectivity depends on the subunit composition of the receptor. There are three NMDAR subunit families: one represented by a single gene (GluN1), the second by four genes (GluN2A-GluN2D) and the third by two genes (GluN3A-GluN3B). NMDARs apart from the Glu recognition sites, have also a polyamine activating regulatory site and separate inhibitory recognition sites for H^+^, magnesium (Mg^2+^) and zinc (Zn^2+^) ions. AMPARs −composed of GluA1-4 subunits− are widespread throughout CNS and mediate fast excitation compared to NMDARs. KARs −composed of GluK1-5 subunits− provide additional contributions to excitatory signaling (Crupi et al. 2019; Karakas et al., 2015).

Ca²⁺ enters via NMDARs and binds to calmodulin (CaM), forming a Ca²⁺−CaM complex. When Ca²⁺ conductance is high, this complex activates Ca²⁺/calmodulin-dependent kinase II (CaMKII) and neuronal nitric oxide synthase (nNOS), the latter binds to PSD-95. Activated nNOS produces nitric oxide (NO) from arginine (Arg); NO then stimulates guanylate cyclase (GC), which converts guanosine triphosphate (GTP) into cyclic guanosine monophosphate (cGMP) (de León-López et al. 2025; Araki et al. 2020). cGMP activates protein kinase G (PKG). Both PKG and CaMKII phosphorylate target proteins, including the AMPAR GluA1 subunit at Ser-831. This modification enables AMPARs, stored in recycling endosomes, to be recruited to the postsynaptic membrane, thereby strengthening the synapse during LTP. However, if Ca²⁺ influx is moderate, Ca^+2^−CaM instead activates protein phosphatase-2B (PP2B/calcineurin), which dephosphorylates GluA1 at Ser845, promoting AMPAR endocytosis and leading to LTD (de León-López et al. 2025).

#### Metabotropic Glutamate Receptors (mGluRs)

Glu also binds to mGluRs which consist of eight cloned heptaspanning receptors grouped into three functional subgroups (Crupi et al. 2019). Group I mGluRs (mGluR1, mGluR5) are located only on postsynaptic neurons and couple to G_q_/G_11_ proteins which activate phospholipase C_β_ (PLC_β_) to produce inositol trisphosphate (IP_3_) and diacylglycerol (DAG). IP_3_ binds to its receptor (IP3R) on smooth endoplasmic reticulum (ER) and mobilizes stored Ca²⁺ increasing excitability and activating CaM (Wang and Zhuo 2012). However, sarco/endoplasmic reticulum Ca²⁺-ATPase 2 (SERCA2) uses the energy from ATP hydrolysis to pump Ca²⁺ from the cytosol back into the ER lumen and lower cytosolic Ca^+ 2^ levels (Britzolaki et al. 2018). DAG activates protein kinase C (PKC) which phosphorylates and activates other kinases from signaling pathways, such as mitogen-activated protein kinase/extracellular receptor kinase (MAPK/ERK), phosphoinositide 3-kinase/protein kinase B (PI3K/Akt) and mammalian target of rapamycin (mTOR). These pathways lead to the translocation of TFs, such as erythroblast transformation specific (ETS)-domain containing protein (Elk), cAMP response element-binding protein (CREB), nuclear factor κ B (NFκΒ) and cellular-Jun/-Fos (c-Jun/c-Fos) −components of activation protein 1 (AP1)−, from the cytosol to the nucleus, where they promote the transcription of genes related to cell survival, neuronal excitability and synaptic plasticity either LTP or LTD based on the receptor and Ca^+ 2^ index (Eddy et al. 2022; Wang and Zhuo 2012).

Group II (mGluR2, mGluR3) and Group III (mGluR4, mGluR6-8) receptors are coupled to G_i/o_ proteins which inhibit adenylate cyclase (AC), the enzyme that converts ATP to cyclic adenosine monophosphate (cAMP). Low cAMP levels lead to PKA deactivation, dephosphorylations and gene repression (Eddy et al. 2022; Manning et al. 2017). Group III receptors are located only on presynaptic neurons where they suppress Glu release. The βγ G-proteins inhibit the VGCCs and activate the G-protein-coupled inwardly rectifying potassium channels (GIRKs) causing hyperpolarization (Rabeh et al. 2023). As a result, the docking and exocytosis of synaptic vesicles is blocked. Group II receptors are expressed both on presynaptic and postsynaptic neurons (Niswender and Conn 2010).

### Glutamate Clearance: The Role of Astrocytes

Glu has a dual role depending on the level of activation. On the one hand, moderate activation of its receptors promotes neuronal growth and survival, providing a trophic effect. Conversely, excessive activation, particularly of extrasynaptic NMDARs, induces a flood of Ca^+ 2^ within the neuron, which in turn injures mitochondria and activates apoptosis, exerting a toxic effect. Therefore, Glu levels must be tightly regulated **(**Dong et al. 2009).

#### Glutamate/Glutamine Cycle

Much of the Glu that is released from nerve terminals is taken up from the extracellular fluid into astrocytic processes by Na^+^-dependent excitatory amino acid transporters (EAATs) 1–5. EAAT1 and EAAT2 are the principal transporters on astrocytes (Magi et al. 2019). In astrocytes, the ATP-requiring glutamine synthetase (GS), in the cytosol, forms glutamine (Gln) from Glu and the toxic NH_3_ which is converted to the nontoxic form ammonium (NH_4_^+^). The conversion of NH_3_ to NH_4_^+^ by GS is critical for both the detoxification of NH_3_ in the brain and nitrogen (N_2_) assimilation. GS is not expressed in neurons.

Gln −not a NT− is then exported to the extracellular fluid and taken up by neurons. Both astrocytes and neurons have sodium-coupled neutral amino acid transporters (SNATs) that concentrate Gln inside the cells. SNAT1/2 are located on neurons, uptake Gln and compose system A (SA), while SNAT3/5 are expressed on astrocytes, mediate both uptake and release of Gln and compose system N (SN). In neurons, Gln is converted back to Glu by phosphate-activated glutaminase (PAG or GLS), a mitochondrial and possibly neuron-specific enzyme in the brain. The trafficking of Glu and Gln between neurons and astrocytes is called the “the Glu/Gln cycle” (Zhang et al. 2024a, b; Magi et al. 2019).

In addition to astrocytes, microglia −CNS-resident immune cells− can also uptake and release Glu (Persson et al. 2006) and express GluRs (Liu et al. 2016) further shaping glutamatergic signaling (Liu et al. 2016; Persson et al. 2006).

#### Lactate (Lac) Shuttle Hypothesis

The uptake of Glu occurs against its concentration gradient, requiring a driving force provided primarily by the Na^+^ electrochemical gradient across the plasma membrane. This process is known as X_AG_^−^ system. Glu enters cells co-transported with three Na^+^ and one H^+^, while one K^+^ is counter-transported. As a result, Glu uptake is electrogenic, causing a net movement of positive charge across the membrane. Since Glu uptake is driven by cation gradients, it represents a secondary active transport process that relies on the Na^+^-K^+^ ATPase. For each Glu molecule uptake, one ATP molecule is consumed to extrude the three Na^+^ in exchange for two K^+^ ions. In addition, conversion of Glu to Gln by ATP-dependent GS consumes another ATP molecule (Zhang et al. 2024a, b; Andersen et al. 2021; Magi et al. 2019).

This energy demand according to the “lactate (Lac) shuttle hypothesis” is met by anaerobic glycolysis, which generates rapidly two ATP molecules per glycose molecule converted to Lac. Astrocyte-derived Lac is transported into neurons via monocarboxylate transporters (MCTs), where is converted into Pyr by lactate dehydrogenase (LDH), driving Krebs cycle (Andersen et al. 2021; Brooks 2018). Importantly, astrocyte-derived Lac is essential for neuronal plasticity and memory formation as it induces the expression of plasticity-related genes such as activity-regulated cytoskeleton-associated protein (Arc), early growth response 1 (Egr1), and brain-derived neurotrophic factor (Bdnf), and enhances NMDAR-mediated intracellular Ca^+ 2^ increase which enables LTP and memory formation (Magistretti and Allaman 2018).

#### Xc^−^ System

Astrocytic metabolism is highly active, which can increase reactive oxygen species (ROS) levels. To counter this, astrocytes rely on the Na⁺-independent, Cl^−^-dependent cystine (Cys₂)/Glu antiporter, known as Xc^−^ system, which mediates a 1:1 exchange of extracellular Cys₂ for intracellular Glu. The heterodimer antiporter is composed of xCT (light chain) and CD98 (heavy chain) (McBean 2002).

Once inside the cytosol, Cys₂ is reduced to cysteine (Cys). Glutamate-cysteine ligase (GCL) by using ATP, joins Glu and Cys to form the dipeptide γ-glutamylcysteine. Subsequently, glutathione synthetase (GSS), also by consuming ATP, adds glycine (Gly) to this dipeptide, to produce γ-glutamylcysteinylglycine or glutathione (GSH). GSH serves as a major antioxidant, protecting cells from free radicals and pro-oxidants. It also functions as a cofactor for various antioxidant and detoxification enzymes, including GSH peroxidases and S-transferases (Lu 2013).

## Immune System: Overview

The immune system defends the body against dangers such as bacteria, viruses and damaged macromolecules. It is divided into innate and adaptive immune system. The innate immune system responds rapidly and non-specifically and relies on dendritic cells (DCs), monocytes/macrophages (MΦ), polymorphonuclear cells (PMNs; neutrophils, basophils, eosinophils), natural killer (NK) cells, and a set of plasma proteins known as the complement system. In contrast, the adaptive immune system, mediated by T and B cells, mounts a slower, but highly specific response. A key feature of adaptive immunity is the formation of immunological memory, which enables a more robust and rapid response upon subsequent exposure to the same threat **(**Wang et al. 2024).

T, B, and NK cells are all lymphocytes with T cells being the most abundant among the others in peripheral blood (Wang et al. 2024). T cells (CD3⁺) are broadly classified into T helper (Th) cells and cytotoxic T lymphocytes (CTLs), distinguished by their expression of CD4 or CD8 T cell receptors (TCRs), respectively. Naïve CD4⁺ T cells can differentiate into specialized subsets with pro-inflammatory, anti-inflammatory, or dual functions. For example, Th1, Th17, and Th22 cells promote inflammation by secreting IFN-γ, tumor necrosis factor α (TNF-α), IL-17 and IL-22. In contrast, Th2 cells exhibit anti-inflammatory properties through the production of IL-4, IL-5, IL-10, and IL-13. Th9 cells, which secrete IL-9, can play either pro-inflammatory or anti-inflammatory roles depending on the disease context (Sun et al. 2023). CTLs and NK cells mediate cytotoxicity by inducing apoptosis of target cells via effector molecules, such as IFN-γ, granzyme B (GnzB), and perforin (Pfn). NK cells are crucial for early cytotoxic responses against viruses and malignant cells (Wang et al. 2024; Sun et al. 2023).

B cells are specialized for Ab production, making them the leaders of humoral immunity. In addition, they can serve as APCs, along with DCs and MΦ. APCs regulate T cell differentiation by forming immunological synapses, where TCRs engage with antigen (Ag) peptide-major histocompatibility complex (MHC) molecules on the APC surface. MHC class I interacts with CD8⁺ T cells, while MHC class II interacts with CD4⁺ T cells. Adhesion molecules and co-stimulatory ligands, such as CD40 and CD40L, further stabilize these interactions. MΦ also play a major role in phagocytosis (Wang et al. 2024).

Finally, PMNs −characterized by their multi-lobed nuclei− phagocytose pathogens, release antimicrobial peptides and enzymes packaged in granules, and migrate rapidly to sites of infection or injury. Neutrophils are the most abundant leukocyte type in peripheral blood and the dominant subset of PMNs (Baggiolini et al. 1978).

## Glutamate and Immune Cells

The discovery that non-neuronal tissues, such as the pancreas and skin, express GluRs (Skerry and Genever 2001) marked a turning point in broadening the understanding of NTs beyond the nervous system, extending their relevance to the immune system as well. Both arms of immunity −innate and adaptive− are influenced by such signaling (Ganor and Levite, 2012) (Table 1).

**Table 1:** Expression and function of glutamate receptors and transporters in immune cells

Immune cell	Glu receptors/transporters	Effects of glu	Key references
T cells	iGluRs (AMPAR, NMDAR), mGluRs (I & III)	GluRs: ↑ Ca²⁺ influx (mGlu1/5R, mGluR2/3 → Kv1.3 channels) AMPAR → chemotactic migration mGluR5 → ↑ cAMP ⊣ Th1 polarization, proliferation or ↑ Ca^+ 2^ → survival? mGluR1 → ↑ ERK1/2 → Th1 polarization, proliferation	Kostanyan et al. (1997) Lombardi et al. (2001) Ganor et al. (2003) Pacheco et al. (2004) Poulopoulou et al. (2005a, 2005b) Poulopoulou et al. (2005a, 2005b) Chiocchetti et al. (2006) Mashkina et al. (2007) Miglio et al., (2005) Zainullina et al. (2011) Orihara et al. (2018)
B cells	Group I mGluRs, KARs	↑ ADAM10 → CD23 cleavage → ↑ IgE & IgG → ↑ humoral immunity	Sturgill et al. (2011)
Natural killer cells	Group I mGluRs, Xc⁻ system	mGluR5 → ↑ IFN-γ, TNF-α, GnzB, Pfn and CXCR4 → ↑ cytotoxicity and chemotactic migration, respectively	Choi et al. (2021) Kim et al. (2023)
Dendritic cells	Xc⁻ system, EAAT1/2	Glu release → modulation of T cell polarization	Pacheco et al., (2006) D’Angelo et al., (2010)
Macrophages	XAG⁻ and Xc⁻ systems	Glu → GSH synthesis	Rimaniol et al. (2000) Rimaniol et al. (2001)
Neutrophils	NMDAR (GluN2B), Xc⁻ system	Glu release → ↑ endothelial permeability NMDAR → ↑ Ca²⁺ → ROS generation Glu → ↑ survival	Collard et al. (2002) Del Arroyo et al. (2019) Kopach et al. (2023)

### Lymphocytes

Kostanyan et al., in 1997, reported first that T cells express GluRs on their plasma membrane (Kostanyan et al. 1997). It is now established that lymphocytes express both iGluRs and mGluRs, which are functionally active and influence multiple aspects of immune performance.

#### iGluRs

Peripheral blood lymphocytes express functional iGluRs. Pre-exposure of lymphocytes to low Glu concentrations enhances Ca²⁺ influx upon stimulation with anti-CD3 monoclonal antibodies (mAbs) or phytohemagglutinin (PHA), thereby activating IL-2 and IL-2 receptor (IL-2R) production (Lombardi et al. 2001). AMPARs are also present on resting T cells and activation of iGluR3s with very low Glu concentrations promotes α_5_β_1_- and α_6_β_1_-integrin-mediated adhesion to the extracellular matrix glycoproteins laminin and fibronectin and C-X-C motif chemokine receptor 4 (CXCR4)-dependent chemotactic migration toward C-X-C motif chemokine ligand 12 (CXCL12), but inhibits IL-10 production (Ganor et al. 2003). However, iGluR3 on activated T cells is cleaved by GnzB (Ganor et al. 2007).

Moreover, the NMDAR-antagonist, 5-methyl-10,11-dihydro-5 H-dibenzo[a, d]cyclohepten-5,10-imine (MK-801), decreases the production of ROS in lymphocytes, whereas elevated NMDA concentrations associate with increased oxidative stress and a subsequent reduction in cell numbers (Mashkina et al. 2007; Tuneva et al. 2003). NMDAR activation inhibits also IFN-γ production in IL-2 treated lymphocytes with T cells being more affected compared to NK cells (Mashkina et al. 2007). These indicate that the level of NMDAR activation plays the same role in cell viability as does in neurons.

Regarding the mechanistic insights into NMDAR-mediated Ca^+ 2^ homeostasis it is known that NMDARs are essential for sustaining Ca²⁺ influx beyond ER store release. Specifically, pre-treatment of T cells with thapsigargin (a SERCA pump inhibitor) causes continuous elevation of cytosolic Ca²⁺ by blocking SERCA-mediated refilling of the ER and promoting extracellular Ca²⁺ entry, whereas MK801 and thapsigagrin together markedly reduce the extracellular influx, underscoring the essential role of NMDARs in maintaining Ca²⁺ entry and proper signaling dynamics. Importantly, NMDAR-dependent Ca²⁺ entry regulates signaling cascades such as MAPK, PI_3_K/Akt, CaMKII, and PKC −kinases critical for T cell proliferation and activation. Accordingly, MK-801 treatment impairs phosphorylation of these kinases in anti-CD3/CD28-stimulated T cells and thus proliferation (Zainullina et al. 2011).

Moreover, NMDAR-1 expression increases upon T cell activation and NMDARs agonists enhance Th2 cytokine secretion, favoring Th2 polarization of CD4^+^ T cells. This can be attributed to the increased sensitivity of Th1 cells to NMDAR-mediated apoptosis via intracellular Ca²⁺ overload (Orihara et al. 2018).

B cells also possess functional KARs, which when activated by KA or Glu, upregulate the expression of disintegrin and metalloprotease 10 (ADAM10). ADAM10 cleaves the low affinity IgE receptor, CD23 (FcεRII), resulting in increased IgE and IgG synthesis. ADAM10 plays a significant role in the enhanced humoral response of Glu-treated B cells, as its inhibition abolishes these stimulatory effects, which appear to occur independently of NMDAR or AMPAR activity (Sturgill et al. 2011).

#### mGluRs

T and B cells express also mGluRs in a Glu concentration-dependent way. Naïve T cells constitutively express mGluR5, whereas mGluR1 expression requires T cell activation via TCR-CD3 complex formation (Pacheco et al. 2004). Resting lymphocytes can also express mRNAs for mGluR2, mGluR3, and mGluR8 (Poulopoulou et al. 2005a, 2005b). However, their signaling cascades in lymphocytes are not fully clarified. First, it was shown that mGluR5 activation in T cells elevates intracellular cAMP levels and consequently suppress T cell proliferation via G_s_/AG pathway, while mGluR1 promotes cell proliferation as it is coupled with MEK-ERK1/2 survival pathways (Pacheco et al. 2004). Later, this was contradicted as it was reported that activation of group I mGluRs elicits a Ca^+ 2^ intracellular increase via G_q_/PLC, inducing c-fos and c-Jun expression which mediate T cell survival and proliferation (Miglio et al., 2005). Group I/II mGluRs drive K⁺ efflux via voltage-gated Kv1.3 channels followed by Ca²⁺ influx (Poulopoulou et al. 2005a, b). Additionally, group I mGluRs activation with low-mid concentration Glu inhibits Fas ligand (FasL) expression, which is part of the apoptotic program called activation-induced cell death (AICD) of activated T cells. This effect is not replicated by iGluRs agonists (Chiocchetti et al. 2006). On the other hand, activation of mGluR5, expressed on cytotoxic NK cells in the liver, by chlorohydroxyphenylglycine (CHPG) induces the production of IFN-γ, TNF-α, GnzB, Pfn. In comparison, mGluR1 expression was not so intense in either cytotoxic or immunoregulatory NK cells which are classified as CD56^dim^ and CD56^bright^, respectively, due to the fluorescence intensity detected by flow cytometry (Choi et al. 2021). Lastly, adipocytes release Glu via the Xc^-^ system and activate CXCR4^+^ NK cells that migrate in visceral adipose tissue, thereby potentiating systemic inflammation (Kim et al. 2023).

### Dendritic Cells (DCs)

Mature DCs release Glu via the Xc^-^ system either upon stimulation with super-antigens like staphylococcal enterotoxin A (SEA) or through both specific and non-specific interactions with T cells. DC-derived Glu activates mGluR5 on T cells in a concentration-dependent manner suppressing the pro-inflammatory Th1 phenotype. This occurs through cAMP- and PKA-dependent activation of C-terminal Src kinase (Csk), which inhibits TCR-mediated PLCγ1 activation and its downstream signaling cascade, ultimately blocking nuclear translocation of NF-κB and nuclear factor of activated T cells (NFAT); both key transcription factors for expression of pro-inflammatory genes, such as IL2, IFNG, TNF, IL6. Conversely, activation of mGluR1s with DC-derived Glu in T cells enhances Th1 phenotype (Pacheco et al. 2006).

Furthermore, lipopolysaccharide (LPS)-induced maturation of DCs upregulates Cys_2_/Glu antiporter chains, resulting in increased Glu and Cys₂ transport. This observation is more prominent in males and older individuals than in females and younger subjects, prompting speculation about age- and sex-related differences. While transcripts for EAAT1 and EAAT2 transporters can be detected, the observed Cl-dependent Glu transport points to Xc^-^ system activity. Moreover, monocytes differentiated into DCs, by IL-4 and granulocyte-macrophage colony-stimulating factor (GM-CSF) in the presence of the Xc^-^ system’s inhibitor L-homocysteic acid (LHC), display reduced expression of DC markers, such as MHC class I/II, CD80, and DC-specific intercellular adhesion molecule-3-grabbing non-integrin (DC-SIGN). However, inhibition of the Xc^-^ system does not impair LPS-induced phenotypic maturation, as expression of MHC class I/II, CD80, CD83, and CD86 is preserved, nor did it affect Ag uptake. While the Xc^-^ system contributes to DC differentiation, it does not in DC maturation (D’Angelo et al. 2010).

### Neutrophils (NEU)

Stimulation of human PMNs with the bacterial peptide N-formyl-methionyl-leucyl-phenylalanine (fMLP), followed by application of their supernatants to endothelial cell cultures, activates endothelial mGluRs, thereby increasing microvascular permeability and promoting NEU transmigration (Collard et al. 2002).

Glu production in NEU can be activated with opsonised *Escherichia coli*, fMLP or Phorbol 12-myristate 13-acetate (PMA), a PKC activator that increases NMDAR activity by reducing Mg²⁺ block. Upon activation, both human primary neutrophils and NEU-like cells upregulate the NMDAR subunit GluN2B, which is induced during systemic inflammation and plays a critical role in NEU maturation and ROS production. Interestingly, the ROS-generating machinery of NEU parallels that of neurons, underscoring shared molecular strategies between the immune and nervous systems. Moreover, tonic extracellular Glu appears to exert protective effects in NEU, similar to neurons, by engaging pro-survival and antioxidant pathways, such as the GSH cycle (Del Arroyo et al. 2019). Importantly, Glu release by NEU can be self-amplifying: extracellular Glu stimulates further Glu secretion, establishing a self-sustaining molecular communication network among NEU (Kopach et al. 2023).

### Macrophages (MΦ)

Glu transport by monocyte-derived ΜΦ (MDMs) is mediated via both X_AG_^-^ (Rimaniol et al. 2000) and X_C_^-^ (Rimaniol et al. 2001) systems with an approximate 4:1 contribution ratio. Both systems cooperate for GSH synthesis which needs Glu and Cys availability. EAATs increase the Glu intracellular pool and thus Cys_2_/Glu antiporter has available Glu to exchange for Cys_2_ (Rimaniol et al. 2001). Notably, TNF-α can increase Glu transport by upregulating the expression of EAATs, highlighting a link between inflammatory signaling and Glu metabolism in MΦ (Rimaniol et al. 2000).

## Alzheimer’s Disease (AD)

Although there are hundreds of neurodegenerative disorders, AD has received the lion’s share of attention (Ballreich et al. 2021; Agarwal et al. 2020). The disease identified first in 1906 by Alois Alzheimer and although several key mechanisms have been identified, the full integration and progression of cellular and intercellular processes remain unclear (Agarwal et al. 2020).

The main histopathological features in AD patients’ brains are the extracellular deposition mainly of amyloid β (Αβ) plaques and/or intracellular neurofibrillary tangles of hyperphosphorylated tau (p-τ). These protein aggregates trigger a cascade of pathological events: mitochondrial dysfunction, increased oxidative stress, impaired autophagy and protein misfolding; all of them ultimately lead to synaptic impairment and cell death especially of cholinergic neurons in the basal forebrain projecting to the hippocampus and, via distinct neuronal populations, to widespread cortical regions including the temporal lobes. Hippocampus and temporal lobes are regions critical for cognitive functions, such as learning, language, and memory formation (Agarwal et al. 2020; Hynd et al. 2004). Cholinergic neurons appear to have impaired ACh production, release and signaling whereas AChE is abundant, resulting in delayed neurotransmission and inhibition of postsynaptic activation (Cáceres et al. 2023; Agarwal et al. 2020). In parallel, NMDAR hyperactivation causes dendritic loss, while chronic glia activation and unresolved inflammation foster neurodegeneration (Cáceres et al. 2023; Agarwal et al. 2020; Hynd et al. 2004). Overall, we have the Αβ, tau, cholinergic, glutamatergic, and neuroinflammation hypotheses (Cáceres et al. 2023; Agarwal et al. 2020).

Nowadays, there are attempts to categorize AD into biological and clinical stages based on biomarkers (e.g., p-τ, αβ) in biological samples (e.g., blood), imaging tests [(e.g., positron emission tomography (PET)] and cognitive symptoms of patients. In summary, AD patients may experience a prolonged asymptomatic phase, with or without αβ deposition. Over time, this progresses to mild-moderate cognitive impairment associated with Aβ and τ pathology, or with Aβ alone, and ultimately culminates in severe cognitive decline with extensive NFTs and αβ plaques (Jack et al. 2024).

Current therapeutic strategies primarily include AChE inhibitors (AChEIs) (e.g., donepezil) and NMDAR antagonists (e.g., memantine) (Agarwal et al. 2020). In recent years, attempts have been made to develop therapeutic mAbs (e.g., aducanumab, lecanemab) that target Aβ epitopes facilitating the clearance of the plaques. Current data from clinical trials show modest cognitive benefits in AD patients (Cummings et al. 2024).

### Glutamatergic Hypothesis

Accumulating evidence suggests that glutamatergic dysregulation and neuronal hyperexcitability are central contributors to the pathophysiology of AD. Functional imaging studies have demonstrated increased hippocampal activity in Aβ-positive individuals with mild cognitive impairment (MCI) during memory tasks. Importantly, these patients exhibit faster cognitive decline compared with Aβ-negative MCI individuals, suggesting that neuronal hyperactivity may precede overt neurodegeneration and symptomatic decline (Hascup et al. 2020). Similarly, hippocampal hyperactivity in cognitively normal older adults has been associated with τ accumulation, further supporting the idea that network dysfunction emerges early during disease progression. Clinical observations of increased seizure susceptibility and epilepsy in AD, particularly during early stages, provide additional evidence for a hyperexcitable neuronal state (Targa Dias Anastacio et al. 2022).

#### Glutamatergic Dysfunction and Excitotoxic Signaling

The glutamatergic hypothesis of AD primarily centers on dysregulation of NMDARs (Cáceres et al. 2023). In the past, human imaging studies with magnetic resonance imaging/spectroscopy (MRI/MRS) and PET have yielded mixed results for NMDARs in AD: depending on the tracer, protocol, and disease stage. Some reports suggested reduced receptor availability or ambiguous signals, whereas others imply increased channel activity or activation states (Hynd et al. 2004). These highlight the limitations of NMDAR-specific ligands and the likelihood of region- and state-dependent effects (Shah et al. 2023).

Particular attention has been given to extrasynaptic NMDARs, which are strongly associated with excitotoxicity (Dong et al. 2009). Memantine, a low-affinity NMDAR antagonist with rapid channel off-rate kinetics, preferentially targets pathological extrasynaptic receptor activity while largely preserving physiological glutamatergic neurotransmission. Nevertheless, the clinical benefits of memantine remain modest and largely symptomatic. If excitotoxicity alone was the primary driver of AD progression, greater therapeutic efficacy might be expected. Instead, memantine is commonly combined with AChEIs to improve attention and arousal rather than substantially restoring long-term memory consolidation. However, several studies report limited additional benefit of combination therapy compared with monotherapy in advanced AD stages (Zuliani et al. 2025).

NMDAR activation also depends on co-agonist binding at the GluN1 subunit, requiring either Gly or D-Ser in addition to Glu binding at GluN2. Elevated D-Ser concentrations have been identified in post-mortem hippocampal and parietal cortex tissue from AD patients, potentially contributing to excessive receptor activation and neuronal hyperexcitability (Orzylowski et al. 2021).

At the molecular level, glutamatergic signaling abnormalities in AD are highly region- and stage-dependent. Post-mortem studies demonstrate altered expression of NMDAR subunits, including GluN1 (NR1) and GluN2A/B, across vulnerable hippocampal and cortical regions, such as the dentate gyrus, cornu ammonis (CA) fields, and entorhinal cortex (Lewerenz and Maher 2015; Hynd et al. 2004). However, findings are inconsistent, with some studies reporting increased receptor expression in selective hippocampal and entorhinal layers, whereas others demonstrate overall receptor downregulation in AD tissue (Yeung et al. 2021; Hynd et al. 2004). These discrepancies likely reflect differences in disease stage, regional pathology burden, and methodological approaches.

Similarly, Glu levels vary across studies. While earlier reports suggested both increases and decreases in glutamatergic signaling (Lewerenz and Maher 2015), a recent meta-analysis indicate an overall reduction in Glu levels in AD patients. This reduction may reflect progressive loss of glutamatergic pyramidal neurons within the hippocampus and cortex (Ebrahimi et al. 2025).

Simultaneously, Aβ and p-τ assemblies enhance further NMDAR signaling through several routes (Yeung et al. 2021; Hynd et al. 2004). Oligomeric Aβ can can potentiate and interact synergistically with polyamines like spermine and synergize with them (Hynd et al. 2004) to intensify the extrasynaptic NMDAR Ca²⁺ magnitude which suppresses CREB signaling while promoting oxidative stress and apoptotic pathways (Lewerenz and Maher 2015).

In parallel, Glu clearance mechanisms become progressively impaired in AD. Aβ impairs astrocytic uptake by reducing EAAT2 expression and by favoring splice variants with poor transport function (Ebrahimi et al. 2025; Castillo-Vazquez et al. 2024); together with potential upregulation of the Xc^-^ system in Aβ-activated microglia, this can elevate extracellular/CSF Glu levels and potentiate excitotoxic drive. In AD preclinical studies, a β-lactam antibiotic, ceftriaxone, upregulates EAAT2 in astrocytes suggesting that restoration of astrocytic Glu transport may represent a therapeutic strategy (Castillo-Vazquez et al. 2024).

Disrupted recycling can push presynaptic neurons toward compensatory amino-acid metabolism: aminotransferase (A-T) pathways that generate Glu appear altered in AD brain, and peripheral A-T patterns (e.g., AST) have been associated with cognitive status in cohorts linking systemic metabolism to central excitotoxic risk (Majid et al. 2025). Both cytosolic and mitochondrial AT levels have been reported to be elevated in the CA1 and CA3 hippocampal regions, as well as in the frontal and temporal cortex (Majid et al. 2025; Liu et al. 2019). Notably, despite evidence of presynaptic stress, VGLUT1 is consistently reduced in AD cortex and hippocampus, suggesting fewer healthy glutamatergic terminals (Mi et al. 2023).

mGluRs add another layer in pathophysiology. Preclinical data indicate that activating the presynaptic group II and III mGluRs can reduce Glu release and dampen excitotoxicity, whereas mGluR5 partners with cellular prion protein (PrP^C^) to form an Aβ-oligomer signaling hub that drives synaptic AMPAR/NMDAR loss (Conway 2020; Liu et al. 2019). Human PET and immunohistochemical studies suggest mGluR5 availability can be decreased in hippocampal/parahippocampal regions in AD, although findings are not entirely uniform across cohorts and methods (Mecca et al. 2020). Lastly, both Aβ and τ perturb PSD-95-anchored complexes, impairing NMDAR and AMPAR trafficking and signaling, amplifying oxidative stress and synaptic failure (Liu et al. 2019; Zhang et al. 2016; Tu et al. 2014).

Glutamatergic dysfunction is also closely linked to metabolic failure as Glu metabolism is highly energy demanding (Gasiorowska et al. 2021; McKenna 2007). In AD, Aβ and τ disrupt mitochondrial function, causing impaired ATP production, ROS accumulation, and altered TCA cycle activity. Consequently, intermediates such as α-KG and OAA are depleted, compromising both synaptic Glu availability and glial clearance capacity. This energetic failure amplifies maladaptive neuroimmune responses (Mohan and Kumar 2025; Eckert et al. 2011).

Finally, neuronal hyperexcitability can be also attributed to impaired GABAergic signaling (Targa Dias Anastacio et al. 2022). GABA is the principal inhibitory neurotransmitter and has been associated with a wide range of behavioral and cognitive functions including learning and memory. GABAergic inhibition coordinates the activity of distinct neuronal populations and limits excessive neuronal firing, thereby maintaining network stability and preventing hyperexcitability. Human studies have reported reduced GABA levels and altered receptor and transporter expression in AD brains (Jiménez-Balado and Eich 2021) which may induce or exacerbate hyperactivity.

### Neuroinflammation Hypothesis

**Fig. 2:**
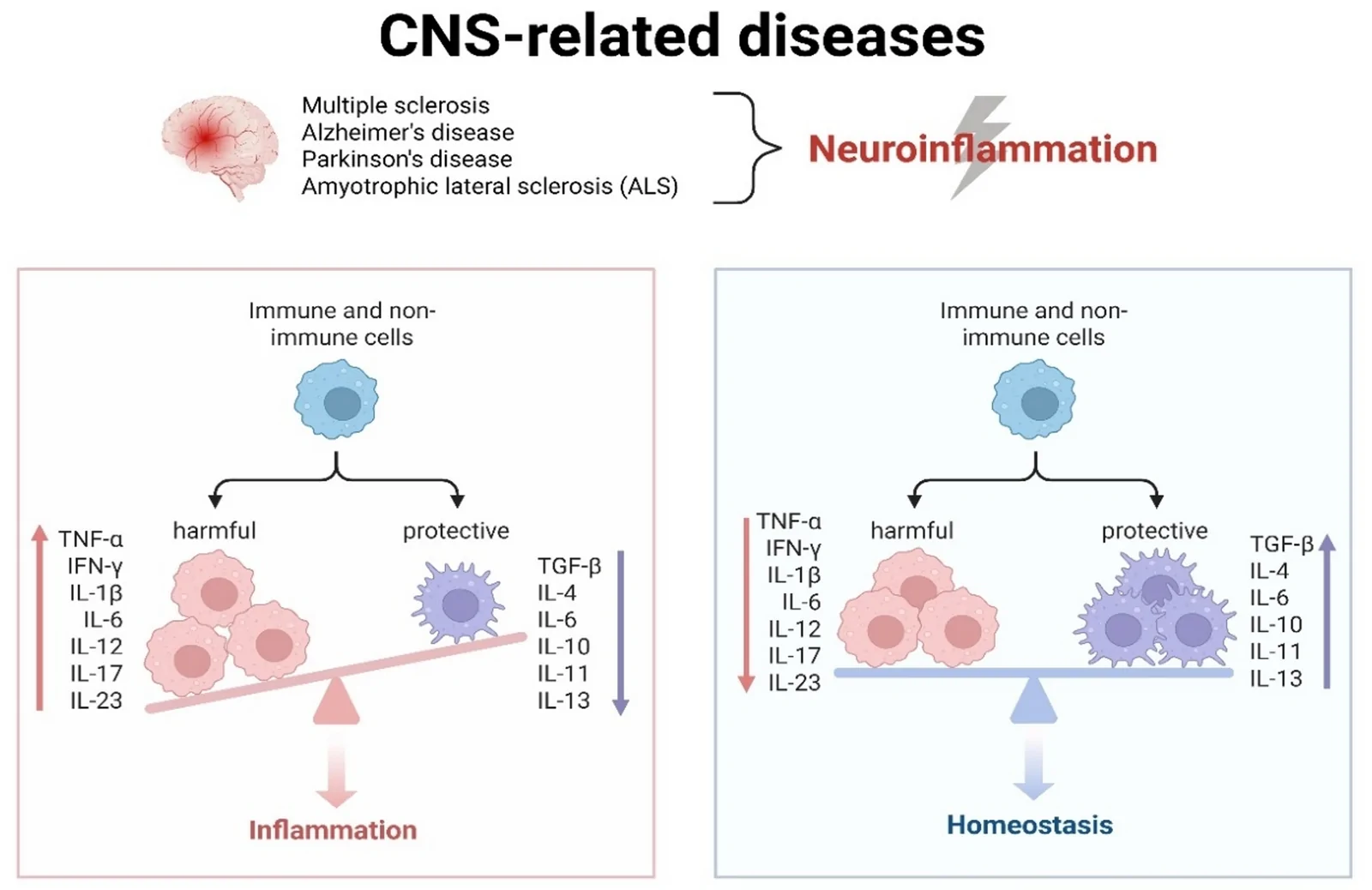
Neuroinflammation. CNS-related disorders, including neurodegenerative diseases such as Alzheimer’s disease, Parkinson’s disease, multiple sclerosis, and amyotrophic lateral sclerosis, share a common hallmark: an imbalance between pro- and anti-inflammatory responses. The pro-inflammatory milieu is characterized by the excessive production of cytokines such as IL-1β, TNF-α, IFN-γ, IL-12, IL-17, and IL-23. In contrast, the anti-inflammatory response is mediated by cytokines including TGF-β, IL-4, IL-10, IL-11 and IL-13. Notably, IL-6 is a pleiotropic cytokine that can exert either pro- or anti-inflammatory properties depending on the microenvironment. Under physiological conditions, a finely tuned balance between these opposing responses maintains CNS homeostasis. *CNS* central nervous system, *IL* interleukin, *TNF* tumor necrosis factor, *IFN* interferon, *TGF* transforming growth factor

CNS-related diseases, such as AD, PD, MS, are characterized by increased inflammation. Astrocytes and microglia appear to be the leaders of a vicious self-perpetuating inflammatory cycle which, in combination with aging, shifts the balance from “protective” toward “harmful” cellular phenotypes (Adamu et al. 2024), as shown in Fig. 2.

#### Astrocyte Dysfunction and Inflammatory Signaling

Astrocytes can be broadly divided into two phenotypes: A1 (pro-inflammatory) and A2 (anti-inflammatory). Post-mortem studies of AD brains propose a predominance of A1 astrocytes. Astrocyte reactivity (astrogliosis) may exist early in disease progression and evolves dynamically over time. While astrocytes contribute to Aβ clearance, their reactive states are heterogeneous and context-dependent, rather than representing a simple transition from an initially protective to a later detrimental phenotype. This reactivity disrupts the BBB, causes neuronal death, and fosters aggregate formation through the secretion of pro-inflammatory mediators such as IL-1β, IFN-γ, and TNF-α, which activate NMDARs, perpetuating neuronal injury, and upregulate β-secretase (BACE) (Leng and Edison 2021), an enzyme critical for the generation of toxic Aβ peptides, including Aβ_1–42_ (Hampel et al. 2021), and with significant diagnostic value (Nicsanu et al. 2022).

#### Microglial Activation and Inflammasome Signaling

Microglial activation is increasingly understood as a spectrum of dynamic and heterogeneous states rather than a binary pro-inflammatory (M1) or anti-inflammatory (M2) phenotype. Recent transcriptomic analyses reveal the presence of diverse disease-associated microglia (DAM) subsets with distinct functional profiles across neurodegenerative conditions. In the context of AD, specific microglial subpopulations exhibit enhanced phagocytic capacity toward Aβ oligomers yet simultaneously promote neuroinflammation and disease progression (Martins-Ferreira et al. 2025).

Microglial responses appear to vary across disease stages. An early anti-inflammatory or protective phase has been proposed during the preclinical stage of AD, followed by a later predominance of pro-inflammatory microglial states during symptomatic disease progression. Initially, microglia may facilitate Aβ clearance and tissue repair; however, chronic stimulation progressively impairs their homeostatic and phagocytic functions (Valiukas et al. 2025).

As specialized macrophages, microglia can internalize Aβ through pattern recognition receptors (PRRs) like toll-like receptors (TLRs) and scavenger receptors (Hanslik et al., 2020; Yu and Ye, 2015). In addition to Aβ, PRRs interaction with DAMPs released by damaged or apoptotic cells is one of the key drivers of inflammation. DAMPs are signals and molecules such as Aβ, high mobility group box 1 protein (HMGB1), cytochrome c, Ca^2+^ ions, and mitochondrial DNA (mtDNA). These DAMPs play a significant role in the activation of microglia and are abundant in the chronically damaged and inflamed state seen in AD, leading further to dysfunctional microglia-microglia communication, which is vital for Aβ clearance (Miao et al. 2023).

Inflammasome activation represents another major mechanism linking neuroinflammation to neurodegeneration. In AD, accumulated Aβ and τ pathology trigger microglial inflammasome activation, ROS production, and cytokine release, which damage neurons. The activation of the NOD-like receptor family, pyrin domain containing 3 (NLRP3) inflammasome leads to the release of protein scaffolds [e.g., apoptosis-associated speck-like protein containing a C-terminal caspase recruitment domain (ASC)] that promote Aβ aggregation. p-τ further impairs the homeostatic surveillance functions of microglia (Leng and Edison 2021; Hanslik et al., 2020).

Currently, microglia serve as a therapeutic target. FDA-approved mAbs Aducanumab, Donanemab, and Lecanemab trigger microglial Fc mediated phagocytosis and have shown some therapeutic efficacy (Valiukas et al. 2025).

#### Glial Crosstalk and Neuronal Injury

Bidirectional communication between astrocytes and microglia is essential for maintaining CNS homeostasis. In AD, dysfunctional astrocyte–microglia signaling contributes to chronic inflammation and impaired Aβ clearance. Alterations in the CD200–CD200R signaling axis, for example, are associated with sustained microglial activation and loss of immune regulation. In parallel, neuron–microglia interactions become disrupted, impairing processes such as neurogenesis, synaptic pruning, and synaptic plasticity (Garland et al. 2022).

#### Peripheral Immune Cell Involvement

As the burden of protein aggregates overwhelms clearance mechanisms, pro-inflammatory cascades intensify and accumulating evidence indicates that AD-associated neuroinflammation is not restricted to resident CNS cells, but also involves extensive participation of peripheral immune populations, both from innate and adaptive immunity (Zhang et al. 2025).

Infiltrating monocytes can enter the brain parenchyma, differentiate into ΜΦ-like cells, and occupy microglial niches, thereby contributing to chronic inflammatory signaling (Leng and Edison 2021). Peripheral monocytes may influence either vulnerability or resilience to cognitive decline, with AD generally associated with a shift toward pro-inflammatory monocyte phenotypes. Several resilience-associated genes, including amyloid precursor protein (APP), have been linked to inflammatory signaling, lipid metabolism, and pathways such as MAPK and IL-1 signaling (Mustafa et al. 2025).

NEU contribute further to neurovascular and inflammatory dysfunction in AD. Both central and peripheral NEU display enhanced inflammatory signaling, neutrophil extracellular trap (NET) formation, and increased adhesion pathway activity, while markers of immature neutrophils are reduced (Aprile et al. 2025). NEU have been identified in the hippocampus and cortex, frequently near the BBB, suggesting a role in vascular injury and BBB disruption. Patients with MCI and AD additionally exhibit impaired NEU phagocytic function (Aprile et al. 2025; Aries and Hensley-McBain 2023). Elevated neutrophil-to-lymphocyte ratio (NLR) has emerged as a potential risk marker associated with increased AD susceptibility across diverse populations, further supporting the contribution of systemic inflammation and NEU-mediated pathways to neurodegeneration (He et al. 2025; Aprile et al. 2025).

Additionally, other innate immune cells also exhibit disease-associated alterations. In MCI, DC-levels correlate with lower CSF Aβ_42_ concentrations, suggesting an association with increased αβ pathology (van Olst et al. 2024). NK cells display stage-dependent functional changes during disease progression. In MCI, NK cells are generally increased in frequency and exhibit a more activated phenotype characterized by enhanced production of GnzB, TNF-α, and IFN-γ, increased C-C chemokine receptor type 7 (CCR7) expression, and reduced inhibitory natural killer group 2 member A (NKG2A) signaling. In contrast, findings in AD dementia are less consistent, with reports describing either reduced NK-cell numbers and cytotoxicity or selective expansion of C-X3-C motif chemokine receptor 1 (CX3CR1+)-NK-cell subsets associated with worse cognitive function (Qi and Liu 2023).

Adaptive immune alterations also emerge early during disease progression, particularly during MCI. CD8 + T cells, especially cytotoxic CD57 + CD8+ terminal effector memory RA T cells (TEMRA), progressively increase across AD stages. Within this population, programmed cell death protein 1 (PD1+)-cells are enriched during MCI and may reflect an activated but partially regulated immune response, whereas PD1- cells become more prominent during AD dementia and are associated with stronger cytotoxic activity and more severe pathology. Reduced PD1 expression also correlates with lower CSF Aβ_42_ levels and higher p-τ concentrations, linking impaired immune regulation to disease severity (van Olst et al. 2024).

In contrast, memory and regulatory CD4 + T-cell populations decline in AD dementia, while senescent CD57 + Killer Cell Lectin-like Receptor G1 (KLRG1)+ CD4 + effector cells increase. Senescent γδ T cells are also elevated and positively correlate with CD8 + TEMRA populations rather than Tregs, suggesting that inflammageing begins early during AD progression (van Olst et al. 2024). Pro-inflammatory CD4 + T-cell subsets, including Th1, Th17, Th22, and Th9 cells, further amplify inflammatory signaling through the secretion of cytokines such as IL-17, IL-23, IL-21, IL-6, IFN-γ, TNF-α, IL-9, and TGF-β1. These mediators reinforce glial activation and promote differentiation of additional naïve CD4 + T cells into pro-inflammatory phenotypes, thereby sustaining chronic immune activation (Zhang et al. 2025).

B cells lastly appear to exert dual and context-dependent effects in AD. Although they can produce Abs against Aβ, they may simultaneously impair microglial function. Naïve-like B-cell populations have been associated with poorer memory performance and increased τ pathology, whereas age-associated B cells (ABCs) correlate with reduced neurodegeneration and lower levels of neuroinflammatory markers, suggesting that specific stages of B-cell maturation may exert protective effects (Zhang et al. 2025).

**Fig. 3:**
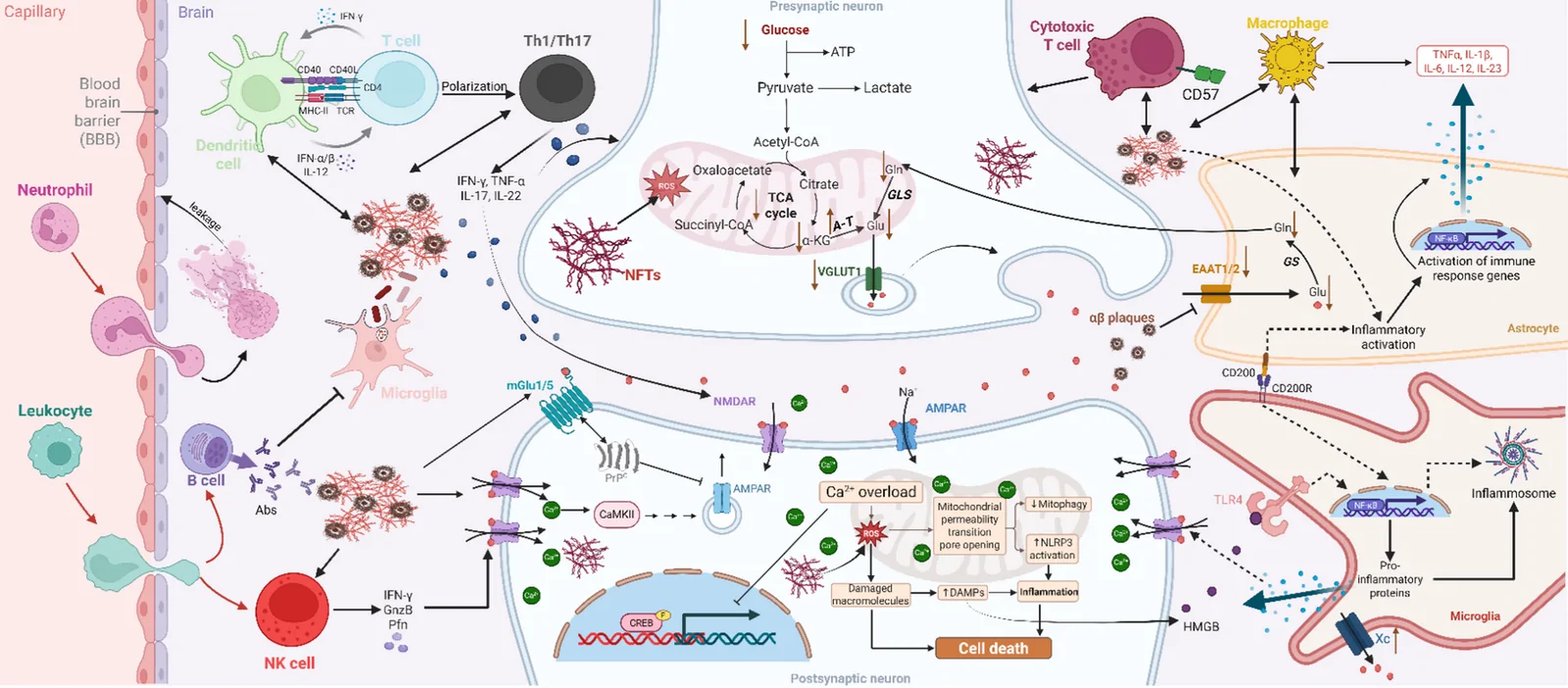
Glutamatergic dysregulation and neuroinflammation in Alzheimer’s disease (AD). Aggregates of Aβ and p-τ initiate interconnected pathological cascades that disrupt neuronal and glial homeostasis, promoting synaptic dysfunction and neurodegeneration. Aβ and τ enhance glutamatergic excitotoxicity through excessive activation of synaptic and extrasynaptic NMDARs, resulting in excessive Ca²⁺ influx. Increased intracellular Ca²⁺ suppresses CREB-mediated neuroprotective signaling and causes mitochondrial dysfunction, ROS accumulation and damaged macromolecule formation, which act like DAMPs (e.g., HMGB), activating inflammatory processes. In parallel, mGluR5–PrP^c^ interactions disturb AMPARs recycling and thus LTP. Altogether, induce neuronal cell death and release of synaptic debris. Presynaptic glutamatergic metabolism is also altered, with impaired glycolysis and TCA-cycle intermediates, dysregulated A-T pathways, altered GLS-dependent Glu production, and reduced VGLUT1 expression contributing to synaptic dysfunction. Astrocytic and microglial dysfunction further exacerbate excitotoxic and inflammatory signaling. Reactive astrocytes display impaired EAAT-mediated Glu uptake and altered GS activity, leading to a dysfunctional “Glu/Gln cycle”. Simultaneously, activated microglia may elevate Glu release through the Xc⁻ antiporter system, thereby potentiating excitotoxic drive. Concurrently, chronic neuroinflammation amplifies neuronal injury through dynamic interactions among astrocytes, microglia, and infiltrating immune cells. Aggregates, DAMPs, ROS, and signals (e.g., cytokines, debris) from neurons activate astrocytic and microglial PRRs (e.g., TLR4) signaling and NLRP3 inflammasome pathways, promoting NFκB-dependent transcription of pro-inflammatory mediators including TNF-α, IL-1β, IL-6, IL-12, IL-17, IL-23, and IFN-γ. Although microglia initially exert protective effects through phagocytosis and clearance of Aβ, chronic activation progressively impairs their phagocytic and homeostatic functions. Also, impaired astrocyte–microglia crosstalk (e.g., CD200–CD200R axis), contributes to sustained inflammatory activation and defective immune regulation. Peripheral immune cell infiltration additionally contribute to AD pathology following BBB disruption. Dendritic cells interact with CD4⁺ T cells through immunological synapse formation involving MHC-II/TCR engagement and co-stimulatory CD40–CD40L signaling, promoting polarization toward pro-inflammatory Th1 and Th17 phenotypes. These T-cell populations release cytokines such as IFN-γ, TNF-α, IL-17, and IL-22, which amplify glial activation and neuronal injury. Cytotoxic CD8⁺ T cells and NK cells further contribute through perforin- and granzyme-mediated cytotoxicity, while neutrophils with NET formation promote vascular dysfunction, and BBB leakage permitting infiltration of more cells. B cells may generate antibodies against Aβ; however, they can also impair microglial phagocytic capacity and contribute to chronic neuroinflammation. *Aβ* amyloid β, *p-τ* phosphorylated tau, *NMDAR* N-methyl-D-aspartate receptor, *CREB* cAMP response element-binding protein, *ROS* reactive oxygen species, *DAMPs* damage-associated molecular patterns, *HMGB1* high mobility group box 1, *mGluR5* metabotropic glutamate receptor 5, *PrPᶜ* cellular prion protein, *AMPAR* α-amino-3-hydroxy-5-methyl-4-isoxazolepropionic acid receptor, *LTP* long-term potentiation, *A-T* aminotransferase, *GLS* glutaminase, *VGLUT1* vesicular glutamate transporter 1, *EAAT1/2* excitatory amino acid transporters 1 and 2, *GS* glutamine synthetase, *Glu/Gln cycle* glutamate–glutamine cycle, *Xc*⁻ *antiporter system* cystine/glutamate antiporter system, *PRRs* pattern recognition receptors, *TLR4* Toll-like receptor 4, *NLRP3* NOD-like receptor family pyrin domain-containing 3, *NF-κB* nuclear factor kappa B, *NFAT* nuclear factor of activated T cells, *TNF-α* tumor necrosis factor α, *IL* interleukin, *IFN* interferon, *CD* cluster of differentiation, *BBB* blood–brain barrier, *MHC-II* major histocompatibility complex class II, *TCR* T cell receptor, *Th1* T helper 1 cells, *NK cells* natural killer cells, *NET* neutrophil extracellular trap

All the above mechanisms underlying the glutamatergic and neuroimmune hypotheses are schematically integrated in Fig. 3.

## AD, Immune Cells and Glutamate: A Speculative Unified Model

Nowadays AD is conceptualized as a progressive systems-level failure rather than a classical proteinopathy driven by αβ or τ aggregation. Within this framework, glutamatergic dysregulation and immune dysfunction may not represent parallel pathological processes, but rather components of a unified neuroimmune network that becomes destabilized during disease evolution. Since immune cells express functional GluRs and transport systems, chronic alterations in extracellular Glu concentrations may directly influence inflammatory behavior within both the CNS and peripheral immune compartments.

A hypothesis is that Glu may behave similarly to ATP: a molecule essential for normal cellular communication that acquires danger-associated properties when persistently elevated extracellularly. This framework may help explain why inflammation persists in AD tissue and the temporal dynamics of neuroimmune responses. Early AD may involve transient or regionally restricted Glu elevations that initially stimulate partially protective immune responses. Over time, however, persistent glutamatergic dysregulation may progressively drive immune exhaustion, impaired phagocytosis, oxidative stress, and chronic cytokine production. In advanced stages, widespread synaptic and neuronal loss may reduce overall glutamatergic signaling, yet localized inflammatory-excitotoxic microenvironments may remain active around damaged synapses, reactive glia, and infiltrating immune cells. Thus, the biological consequences of Glu dysregulation may exhaust protective immune responses while maintaining inflammatory signaling.

Such a transition could have major implications for microglia. In early disease stages, microglia exposed to modest increases in extracellular Glu may initially enhance synaptic surveillance and Aβ clearance. However, prolonged stimulation may induce progressive metabolic stress, mitochondrial dysfunction, and oxidative damage, ultimately shifting microglia toward chronically reactive yet dysfunctional states. Therefore, glutamatergic dysregulation may not merely activate microglia but fundamentally alter their long-term functional trajectory. Another possibility is that activated microglia may express higher levels of GluRs compared to resting microglia and these receptors may be coupled to inflammatory processes. They can even induce inflammation indirectly, if their general effects of intracellular Ca^+ 2^ and cAMP converge into hallmark transcriptional regulators of inflammation (e.g., NFκB, MAPK).

Astrocytes may represent another central node linking Glu imbalance to neuroinflammation. Since astrocytes are responsible for maintaining extracellular Glu homeostasis, even subtle disturbances in their metabolic or transporter function could amplify inflammatory cascades. Chronic inflammatory exposure may progressively reduce the ability of astrocytes to buffer extracellular Glu, while simultaneously increasing their secretion of cytokines and chemokines. This creates the possibility of a self-perpetuating cycle in which inflammatory mediators impair Glu clearance, whereas elevated extracellular Glu further sustains astrocytic and microglial activation. In this scenario, astrocytes transition from protective metabolic supporters into amplifiers of inflammatory excitotoxic stress.

Another speculative possibility is that prolonged glutamatergic stimulation promotes a shift toward cytotoxic and senescent immune phenotypes characteristic of inflammageing. Persistent receptor activation may induce chronic Ca²⁺ signaling, oxidative stress, and metabolic remodeling in immune cells, gradually favoring Th1, Th17, CTLs, NK and plasma cell responses over regulatory or reparative phenotypes including Tregs and Th2. Such mechanisms could contribute to the increasing inflammatory burden observed during disease progression. Mediators released by these cells may further disrupt neuronal and glial glutamatergic signaling and even activate the complement system, reinforcing damage.

NEU may be particularly relevant in this context because they appear capable of both responding to and releasing Glu. Their accumulation near the BBB in AD raises the possibility that Glu contributes directly to neurovascular dysfunction. Extracellular Glu may increase endothelial permeability and facilitate leukocyte transmigration, while infiltrating neutrophils release inflammatory mediators that further destabilize BBB integrity. Thus, glutamatergic dysregulation may indirectly accelerate peripheral immune infiltration into the CNS.

**Fig. 4:**
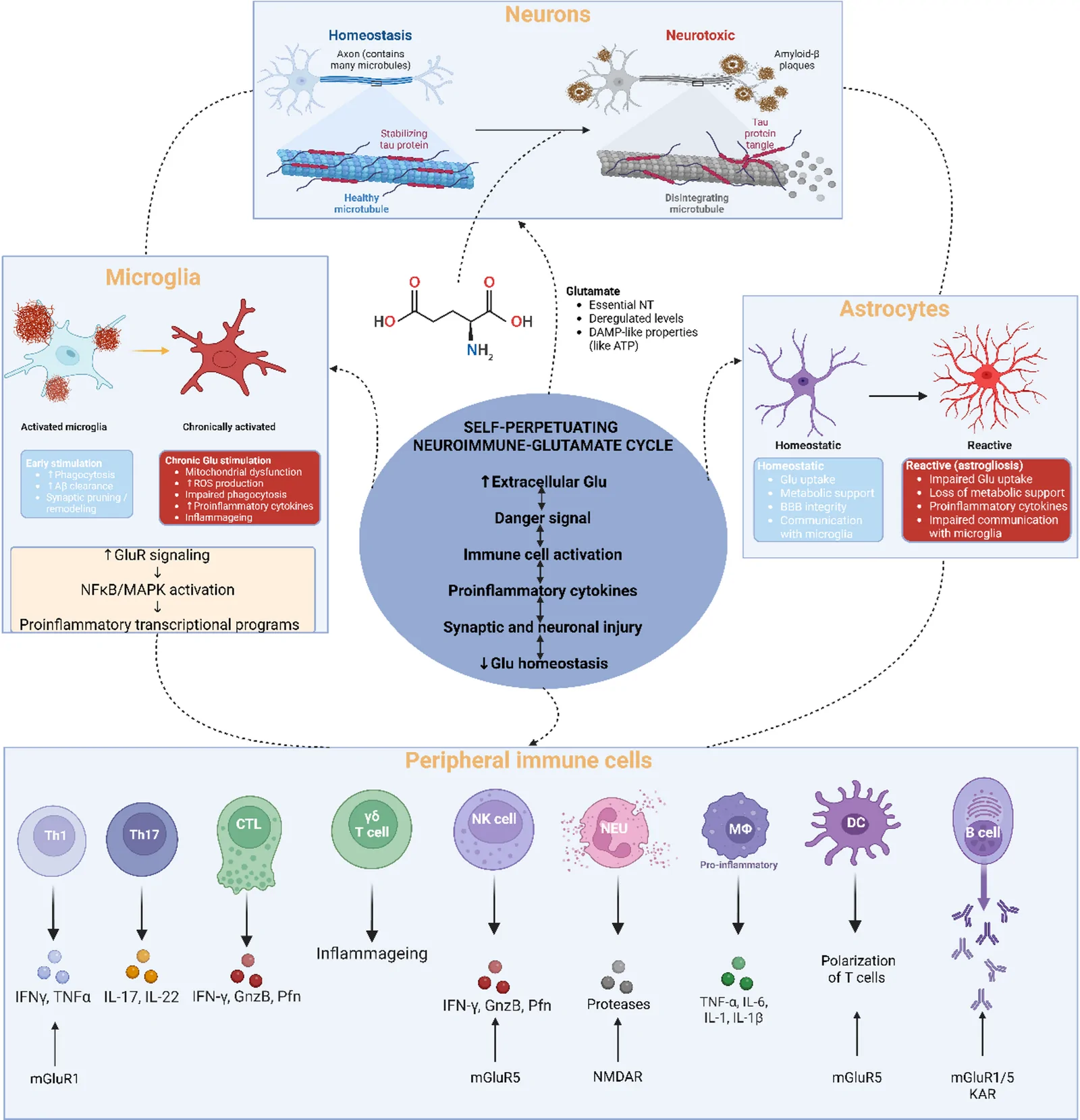
A unified neuroimmune–glutamate (Glu) model in Alzheimer’s disease (AD). This speculative model illustrates how chronic glutamatergic dysregulation may drive a self-amplifying interaction between neurons, glial cells, and peripheral immune populations during AD progression. Persistently elevated extracellular Glu potentially acts as a danger-associated signal that promotes a cycle of immune-cell activation, synaptic injury, and disruption of Glu homeostasis. Early immune cell activation may initially enhance aggregate clearance, and tissue remodeling; however, chronic Glu exposure progressively induces sustained inflammageing and tissue damage where cells lose their homeostatic functions and adapt to the environment becoming themselves inflammatory. Peripheral immune cells reinforce this cycle. The deregulated Glu homeostasis enhances more the adoption of proinflammatory phenotypes. For example, dendritic cells upon mGluR1 stimulation polarize T cells towards Th1 phenotype. Th1 cells and NK cells cytotoxic activity is further promoted upon mGluR5 signaling while humoral immunity can be facilitated through mGluR- and KAR signaling. Lastly, neutrophils release proteases after NMDAR activation. Collectively, these mechanisms support a model in which chronic Glu imbalance, glial dysfunction, inflammageing, and peripheral immune activation form a feed-forward loop that sustains neurodegeneration. *mGluR* metabotropic glutamate receptor, *KAR* kainite receptor, *NMDAR* N-methyl-D-aspartate receptor

Figure 4 depicts a speculative unified neuroimmune–glutamate framework model.

## Conclusion

Glu beyond neurotransmission, functions also as an immunotransmitter, shaping immune cell phenotypes, while dysregulated Glu signaling can link protein aggregation, glial reactivity, peripheral immune infiltration, and synaptic failure into a pathological network in AD. These propose Glu as a bridge between immunity and neurodegeneration.

Therapeutically, combining classical neuroprotective strategies, such as selective NMDAR modulation or EAAT2 upregulation, with immune-targeted glutamatergic interventions (e.g., mGluR5 regulation or Xc^−^ system modulation in glia and leukocytes) offers a promising multi-targeted approach. Integrating these strategies with current immunotherapies, including monoclonal antibodies against amyloid plaques, could address both protein aggregation and neuroimmune-excitotoxic pathways, potentially enhancing clinical outcomes. While not available in all countries, mAbs targeting amyloid plaque clearance have shown modest disease-modifying effects, reducing amyloid burden and slowing cognitive decline in select patient populations. However, their impact is limited, and they do not fully address neuroinflammation, glutamatergic dysregulation, or tau pathology, highlighting the need for multitargeted approaches.

On the other hand, biomarker development may focus on CSF or plasma Glu/Gln ratios, imaging of mGluR5 or EAAT2 availability, and immune transcriptomic signatures reflecting glutamatergic modulation. Longitudinal studies combining these biomarkers with imaging and cognitive assessments could enable patient stratification and guide precision interventions. Additionally, exploring sex- and age-related differences in Glu-immune signaling may uncover underappreciated determinants of AD susceptibility and progression. Lastly, there is need of evidence on microglia-Glu signaling in AD. Microglia influence neuroinflammation, synaptic dysfunction, and amyloid or tau pathology. However, there is no mechanistic data in humans, and the in vitro generation of *bona fide* human microglia remains challenging mainly due to the lack of well-defined surface and intracellular markers.

While these translational approaches remain exploratory, integrating high-resolution human tissue studies, in vivo imaging, metabolic profiling, and immunogenomic analyses could clarify receptor- and cell-specific contributions of Glu signaling.

## Data Availability

No datasets were generated or analysed during the current study.
